# Storage Stability of *Brettanomyces bruxellensis*-Spoiled Pinot Noir After UV-C Treatment

**DOI:** 10.3390/foods14183164

**Published:** 2025-09-11

**Authors:** Svetlana Cvetkova, Elke Herrmann, Benedikt Woll, Mario R. Stahl, Dominik Durner, Maren Scharfenberger-Schmeer

**Affiliations:** 1 Weincampus Neustadt, Dienstleistungszentrum Ländlicher Raum (DLR) Rheinpfalz, Breitenweg 71, 67435 Neustadt an der Weinstraße, Germanydominik.durner@hs-kl.de (D.D.); 2 Department of Food Technology and Bioprocess Engineering, Max Rubner-Institut, Federal Research Institute of Nutrition and Food, Haid-und-Neu-Straße 9, 76131 Karlsruhe, Germanymario.stahl@mri.bund.de (M.R.S.)

**Keywords:** UV-C, *Brettanomyces bruxellensis*, wine, phenols, volatile esters, 4-ethylguaiacol

## Abstract

The application of ultraviolet-C (UV-C) light has emerged as a promising non-thermal alternative to chemical preservatives in winemaking. This study investigates the efficacy of UV-C treatment on the microbial stability of Pinot noir wine during a 12-week storage period at 20 °C, with a focus on the spoilage yeast *Brettanomyces bruxellensis*. Microbiological analysis demonstrated complete and sustained inactivation of 10^5^ CFU/mL *Brettanomyces bruxellensis* after UV-C treatment with no detectable regrowth during the 12-week storage period. Untreated wine showed 1-log increase in *Brettanomyces bruxellensis* during the 12-week storage period and significant production of volatile esters and 4-ethylguaiacol. At the same time, a significant reduction in coumaric acid concentration was determined and attributed to *Brettanomyces bruxellensis* metabolism. UV-C-treated wine showed marginal increases in 4-ethylguaiacol attributed to the residual activity of *Brettanomyces bruxellensis* after UV-C treatment. Volatile esters did not significantly change during the 12-week storage period. The findings of this study demonstrate that UV-C treatment can ensure the microbiological storage stability of red wine.

## 1. Introduction

Established thousands of years ago and rich in history, wine stands as one of the most enduring and widely appreciated fermented beverages, revered not only for its complex and distinctive sensory attributes but also for its profound cultural and historical significance across civilizations [1]. The fermentation process, in the vast majority of cases driven by *Saccharomyces cerevisiae*, is a central step to transforming must into wine and forming its chemical and sensory profile. While *Saccharomyces cerevisiae* and sometimes other non-Saccaromyces species are essential for successful fermentation and positive aroma development, others can be detrimental. Spoilage microorganisms may impair product quality by producing off-flavors, reducing shelf-life, and challenging product safety [2]. *Brettanomyces bruxellensis* is one of the most severe spoilage microorganisms during wine production and is probably the most well-adapted yeast species to dry red wine [3]. Belonging to the *Pichiaceae* family, *Brettanomyces bruxellensis* has been extensively studied in oenology. This yeast shows a high tolerance to ethanol, can survive at low pH, and limited oxygen availability, and is able to metabolize xylose, conditions typical of finished wines in barrels [4,5]. It is ubiquitously found in wineries around the world and is infamous for its ability to produce volatile phenols such as 4-ethylphenol, 4-ethylguaiacol, and 4-ethylcatechol [6,7]. *Brettanomyces bruxellensis* transform hydroxycinnamic acids into volatile phenols in wine via a two-step enzymatic process. Studies show that hydroxycinnamic acids such as p-coumaric acid, ferulic acid, and caffeic acid are decarboxylated by cinnamate decarboxylase. This produces vinyl derivatives, which are then reduced by vinylphenol reductase to 4-ethylphenol and 4-ethylguaiacol [6,8]. These compounds contribute to undesirable aroma attributes often described as horse sweat, leather, smoky or pharmaceutical [9,10]. Cell counts of *Brettanomyces bruxellensis* in the range of 10^5^ to 10^6^ CFU/mL have been shown to significantly influence wine quality within a few weeks through the formation of volatile compounds [11]. Therefore, controlling the growth and metabolic activity of this yeast is essential to maintain the quality and stability of wine during storage.

Sulfur dioxide (SO_2_) traditionally serves as the main antimicrobial and antioxidative agent in winemaking. Its multiple functions make SO_2_ highly effective for preventing spoilage and oxidative degradation [12,13]. Nevertheless, rising temperatures as a result of climate change have led to wines containing less acid, resulting in higher pH [14]. This reduces the antimicrobial effect of SO_2_, favoring the growth of spoilage microorganisms and necessitating significantly higher SO_2_ additions [15]. Health concerns further complicate the use of sulfites in food products. In 2022, the European Food Safety Authority (EFSA) published findings suggesting that sulfite intake, especially for sensitive individuals or in cases of cumulative intake from various dietary sources, may result in adverse health effects such as respiratory reactions [16]. Several other alternative, non-chemical techniques exist for the microbial stabilization of wine. These include filtration, thermal processes such as flash pasteurization [17], and non-thermal treatments such as Ultra-High-Pressure Homogenization (UHPH) [18,19,20,21] and Pulsed Light (PL) [22,23,24,25]. While effective to varying degrees, these methods often have some practical limitations. Flash pasteurization, for example, may negatively influence sensory quality by altering the wine’s flavor profile [26], while certain filtration methods may strip the wine of color and phenolic content, thus impairing authenticity [27]. This has all led to increased pressure on the wine industry to develop effective yet non-chemical preservation methods.

Ultraviolet-C (UV-C) irradiation has been shown as a promising non-thermal, chemical-free technique capable of achieving microbial inactivation of several food and beverage products. UV-C light, operating in the wavelength range of 200–280 nm, induces DNA damage in microbial cells, leading to replication inhibition and, eventually, cell death [28,29]. UV-C treatment was officially recognized as a safe food processing technique by the U.S. Food and Drug Administration (FDA) in 2000 and remains approved under 21 CFR §179.39 [30]. Regulatory acceptance has extended internationally, with approvals granted by Health Canada (2004) and the European Union (EU Regulation 2017/2470) [31] for use in the preservation of various beverages, including fruit juices, cider, and milk. Its germicidal efficacy has been well documented across a variety of food and beverage products. In particular, studies have demonstrated that UV-C treatments effectively reduce the levels of spoilage microorganisms such as *Escherichia coli K12*, *Salmonella typhimurium*, *Saccharomyces cerevisiae*, *Brettanomyces bruxellensis*, *Lactobacillus plantarum*, *Alicyclobacillus acidoterrestris*, *Acetobacter aceti*, *Metschnikowia pulcherrima*, and more in fruit juices, including grape juice and wine, with minimal negative effects on antioxidant content, polyphenols, and sensory attributes [32,33,34,35,36,37,38,39]. The studies showed that effects, such as absorption, cell count, and the type of harmful microorganism can significantly influence the efficiency of the microbial stabilization of wine via UV-C treatment [38,40,41]. The study by Cvetkova et al. [42] showed that UV-C treatment with doses above microbiologically required doses can promote chemical changes with negative effects on the sensory properties of wine, such as the formation of 2-aminoacetophenone, a decrease in monoterpene and C13-norisoprenoid levels, and a decrease in phenol levels. However, a long-term study by Junqua et al. [35] showed no significant changes in the sensory and chemical properties of wine 20 months after UV-C treatment. A study by Hirt et al. [43] showed that the effectiveness of UV-C treatment depends on the flow conditions in the reactor. Turbulent flow conditions have the greatest inactivation efficiency, whereas laminar flow conditions are in general less effective. For highly absorbent liquids (optical density (OD) > 20), guided laminar flow with flow guiding elements (FGEs) was equally effective as turbulent flow conditions. Hirt et al. described the inactivation of *Saccharomyces cerevisiae* under different flow conditions in UV-C reactors using the Weibull model, which allows the inactivation process to be predicted as a function of flow type and absorption.

The efficiency of UV-C treatment is often investigated immediately after its application in wine. However, the preservative effects of UV-C treatment during extended periods of wine storage remain largely unexplored. This study aims to evaluate the preservative effects of UV-C treatment on the microbial stability and chemical integrity of red wine with regards to the spoilage yeast *Brettanomyces bruxellensis* and the development of its cell count over time. For verification of the microbiology results, a focus of the study was on the *Brettanomyces bruxellensis*-specific secondary metabolite 4-ethylguaiacol as well as ethyl and acetate esters as unspecific secondary metabolites. The key precursor substance of *Brettanomyces bruxellensis*-specific secondary metabolism, p-coumaric acid, was analyzed to evaluate the potential of *Brettanomyces bruxellensis* to contribute to changes in the phenolic profile of wine. Additionally, caftaric acid was quantified as an oxidation marker to assess the extent of UV-C-induced oxidation after UV-C treatment.

## 2. Materials and Methods

### 2.1. Materials

Yeast extract peptone dextrose, cycloheximide, yeast extract peptone mucilage, Man–Rogosa–Sharpe, chloramphenicol, and kanamycin were purchased from Carl Roth (Karlsruhe, Germany); ethanol (≥96%) was purchased from Berkel AHK (Ludwigshafen, Germany); gallic acid, caftaric acid, coumaric acid, and catechin were purchased from Merck (Darmstadt, Germany); and potassium hydrogen phosphate (≥99.5%) and phosphoric acid (≥85%) were purchased from ORG Laborchemie GmbH (Bunde, Germany). All chemicals were of analytical reagent grade as a minimum. HPLC-grade water was obtained from a Milli-Q system (Purelab flex 4, Veolia Water Technology GmbH, Celle, Germany).

### 2.2. Wine

Wine was produced using *Vitis vinifera* L. cv. Pinot noir grapes. The grapes were harvested from the experimental vineyards of Dienstleistungszentrum Ländlicher Raum Rheinpfalz (Neustadt an der Weinstraße, Germany) in the 2021 vintage. After harvesting, the grapes were destemmed and crushed. Subsequent maceration lasted for 12 days in 100 L stainless steel fermenters. The must was inoculated with *Saccharomyces cerevisiae* (Lalvin CY 3079 YSEO; Lallemand, Montreal, QC, Canada), fermented at a constant temperature of 25 °C ± 2 °C up to a residual sugar content of <1 g/L. The wine was racked from the yeast lees, and after two weeks, it was inoculated with *Oenococcus oeni* (Lalvin VP41; Lallemand, Montreal, QC, Canada). Malolactic fermentation was completed after three weeks. Afterwards, the wine was filtered with a sheet filter (BECO-COMPACT PLATE A400 SF/ASF; Eaton, Dublin, Ireland) using 0.4 µm filter sheets (BECO sterile 40; Eaton, Dublin, Ireland) and stabilized with 25 mg/L free SO_2_ (solution sulfureuse P15; Erbslöh, Geisenheim, Germany). The wine chemical data was pH 3.5, 13.0% vol. Alc., 0.7 g/L residual sugar, 4.0 g/L tartaric acid, 0.1 g/L malic acid, 2.6 g/L lactic acid, and 0.47 g/L volatile acidity. The wine was stored at 12 °C ± 1 °C in a 50 L sterile stainless steel keg pressurized at ~1.4 bar with liquid nitrogen for four months until the start of the experiments.

### 2.3. Inoculation of Wine with Brettanomyces bruxellensis

Four days before the start of the experiments, an ethanol tolerant *Brettanomyces bruxellensis* strain (internal strain number: Y191; provided from winery Köninger, Kappelrodeck, Germany; Chardonnay, 2018) was cultivated for 72 h in yeast extract peptone dextrose (YPD) nutrient broth at pH 6.5 on a shaking incubator (ZWY-1102C; Labwit Scientific, Melbourne, Australia) at 90 rpm. The adaptation of *Brettanomyces bruxellensis* to high ethanol concentration in YPD was carried out according to the method previously described by Cvetkova et al. [44]. The inoculation of *Brettanomyces bruxellensis* was performed at a cell count of 10^5^ CFU/mL. UV-C treatment was carried out 10 min after inoculation.

### 2.4. Experimental Setup

One week before the start of the experiments, the Pinot noir wine was sterile filtered using a bottle cap sheet filter (pore size 0.45 µm, SFCA-membrane; Thermo Scientific, Waltham, MA, USA). After filtration, the wine had an absorption of 24 AU ± 0.7 AU at 254 nm. After filtration, a 5 mL sample was taken and tested for sterility by plating for the presence of bacteria and yeast. Determination took place in triplicate. The wine was divided into three batches subjected to three different treatments: (1) ‘Control’: wine without *Brettanomyces bruxellensis* inoculation and without UV-C treatment; (2) ‘UV-C untreated’: wine with *Brettanomyces bruxellensis* inoculation (cell count: 10^5^ CFU/mL) and without UV-C treatment; (3) ‘UV-C treated’: wine with *Brettanomyces bruxellensis* inoculation (cell count: 10^5^ CFU/mL) and with UV-C treatment. Cvetkova et al. [44] showed that the Weibull model can be applied to describe the inactivation of *Brettanomyces bruxellensis* in wine, and therefore, the Weibull model was used to determine the microbial-relevant UV-C dose. Hirt et al. [43] revealed the relation between the wine absorption and inactivation kinetics. FDA Regulation 21 CFR 179 [30] specifies that 5-log inactivation must be reached for microbial stability after UV-C treatment. Based on these considerations and based on the given wine absorption and the inoculated cell count, a UV-C dose of 2.4 kJ/L was determined for the experiments to reach 5-log inactivation. After treatment, all batches were subjected to 12 weeks of storage at 20 °C ± 1 °C in a dark room. Wine samples were stored at 20 °C in the dark to reflect standard ambient conditions commonly encountered in wine storage and laboratory settings. Storage in the dark was applied to avoid photochemical reactions and ensure that any observed changes were due to UV-C treatment or microbial metabolism rather than light exposure. All experiments were carried out in triplicate. The sampling for microbial analysis was performed every two weeks for all batches. Chemical analyses were conducted after completion of the storage time.

### 2.5. UV-C System and Dose

The UV-C treatment was performed at 254 nm, in a pilot-scale thin-film UV reactor, built and put into operation by the Institute for Food and Bioprocess Engineering of the Max Rubner-Institute (Karlsruhe, Germany). The reactor consisted of five identical units each equipped with a 20 W low-pressure mercury lamp (UVPro FMD Series; Bioclimatic B.V., Nieuw-Vennep, Netherlands) and seven fluid guiding elements (FGEs) for flow control. The length of each FGE was 60 mm. The five units of the reactor were connected with standard thermoplastic soft polyvinyl chloride (PVC) tubes (4.8 mm I.D., 9.8 mm O.D.; Heidolph, Schwabach, Germany). A peristaltic pump (Hei-FLOW Precision 06; Heidolph, Schwabach, Germany) was used at a flow rate of 100 L/h ± 1 L/h. A cooling system (Julabo F30VC/3; Seelbach, Germany) was included to keep the temperature in the reactor constant at 20 °C ± 1.0 °C. The processing of UV-C treatment was carried out according to a published method by Hirt et al. [43]. The UV-C dose was controlled via the chemical actinometry method of Rahn [45].

### 2.6. Determination of Cell Counts of Brettanomyces bruxellensis

All samples were prepared as ten-fold dilution series in 0.9% NaCl. All dilutions were plated within one hour after sampling on YPD agar with antibiotics (25 μg/mL kanamycin sulphate and 30 μg/mL chloramphenicol) in triplicate. Additionally, 1 mL of each undiluted sample was plated in triplicate on yeast extract peptone mucilage (YPM) with cycloheximide (50 µg/mL) and Man–Rogosa–Sharpe (MRS) with cycloheximide (50 µg/mL) agar to ensure that no contamination of lactic acid and acetic acid bacteria during the experiments took place. The YPD, YPM and MRS agar plates were incubated at 30 °C ± 2 °C for 48 h. After incubation, colonies were counted.

### 2.7. Photometric Measurement of Wine

The absorption of wine at 254 nm was measured with a UV-Vis double-beam photometer (V-740; Jasco, Tokyo, Japan). The samples were measured in 10 mm pathlength micro-UV cuvettes (Brand, Wertheim, Germany).

### 2.8. GC-MS with SIDA Analysis of Volatile Compounds

The analysis of 4-ethylguaiacol was performed according to the method described by Schober et al. [46], using gas chromatography coupled to a quadrupole mass spectrometer (GC-qMS). The analytical system consisted of an AS 3000 autosampler, a Trace GC gas chromatograph, and a Trace MS quadrupole mass spectrometer (all Thermo Fisher Scientific, Waltham, MA, USA). Sample injection was carried out using the concurrent solvent recondensation large-volume splitless injection technique according to Magni and Porzano [47], allowing injection volumes of up to 50 µL while minimizing sample loss. Separation was performed on a fused silica capillary column (30 m × 0.25 mm i.d., 0.5 µm polyethylene glycol coating; Phenomenex, Torrance, CA, USA), connected via a deactivated pre-column (4 m × 0.53 mm i.d.; BGB Analytik AG, Rheinfelden, Switzerland). Helium was used as the carrier gas under constant pressure (75 kPa). The GC oven was programmed from 47 °C (8 min hold), increased at 5 °C/min to 240 °C (15 min hold). The mass spectrometer was operated in electron impact (EI) mode at 70 eV, in full scan mode (m/z 29–300), with the ion source temperature set to 230 °C. Quantification of 4-ethylguaiacol was based on extracted ion traces and performed using a 5-point external calibration covering a concentration range from 10 µg/L to 1000 µg/L. Calibration curves showed linearity with an R^2^ ≥ 0.97.

### 2.9. HPLC Analysis of Phenols

The quantification of catechin, gallic acid, caftaric acid, ferulic acid, and coumaric acid was performed using the method described by Golombek [48], with minor modifications. Sample analysis was conducted using a high-performance liquid chromatography (HPLC) device (Jasco, Tokyo, Japan) equipped with a diode array detector (DAD) (MD-4010; Jasco, Tokyo, Japan). An injection volume of 10 µL was employed at a constant flow rate of 1.0 mL/min. Chromatographic separation was achieved using a Gemini NX C18 column (150 mm × 4.5 mm, 3 µm particle size; Phenomenex, Torrance, CA, USA), maintained at a temperature of 25 °C. The analysis was conducted using a gradient elution protocol. The eluent A was linearly decreased from 100% to 88% over 12 min, followed by an isocratic hold at 88% for 6 min. Subsequently, eluent A was further reduced to 64% over the next 12 min and then rapidly decreased to 25% within 1 min. This was followed by a 3 min isocratic hold at 25%. Finally, the gradient returned to 98% eluent A over 2 min to re-equilibrate the column. Eluant A consisted of acetonitrile and phosphate buffer (5:95, *v*/*v*), while the other eluant was composed of acetonitrile and phosphate buffer (50:50, *v*/*v*). The phosphate buffer was prepared with 10 mmol/L KH_2_PO_4_ and adjusted to pH 1.5 using 85% H_3_PO_4_. Calibration was performed using an external calibration method.

### 2.10. Statistical Data Analysis

The statistical analysis was performed using XLSTAT (Version 2021.2.2.1147 (32-bit), Addinsoft SARL, Paris, France). The normality of discrete data was tested using the Shapiro–Wilk method (*p* ≤ 0.05). Fisher’s letters indicate difference (LSD) post hoc test (*p* ≤ 0.05) was applied. Chemical data was evaluated for significant differences between batches using one-way analysis of variance including interactions (ANOVA, *p* ≤ 0.05).

## 3. Results

### 3.1. Microbiological Stability of Pinot Noir During 12-Week Storage

Table 1 shows the cell counts of *Brettanomyces bruxellensis* in Pinot noir wine over a 12-week storage period subjected to different treatments. In the UV-C-treated wine and in the control, *Brettanomyces bruxellensis* remained consistently below the limit of quantification (LOQ) during the storage period of 12 weeks. The findings confirm the application of the UV-C technique as a reliable non-thermal preservation method for ensuring microbial stability in wine over prolonged storage periods. UV-C treatment causes the formation of pyrimidine dimers in DNA, which leads to DNA damage. In turn, this inhibits the reproduction processes of microorganisms, which leads to inactivation of the microorganism and thereafter to cell death [28,29]. These results are consistent with previous findings in other UV-C-treated beverages such as orange and lemon–melon juice, wherein no microbial growth was detected during storage [33,49,50]. Conversely, the UV-C untreated wine showed a statistically significant increase in cell counts (*p* < 0.0001), with a peak at week 8 (1.4 × 10^6^ ± 4.0 × 10^4^ CFU/mL) followed by a decrease until week 12. The decrease in *Brettanomyces bruxellensis* cell counts in the UV-C-untreated Pinot noir wine between weeks 8 and 12 suggests the onset of the stationary or early death phase, marking the transition toward the end of the microbial growth cycle [51]. The growth of *Brettanomyces bruxellensis* in UV-C-untreated Pinot noir, corresponding to a ~1-log increase, highlights the risk of increasing spoilage of wine during storage without treatment.

### 3.2. Volatile Compounds

**Figure 1:**
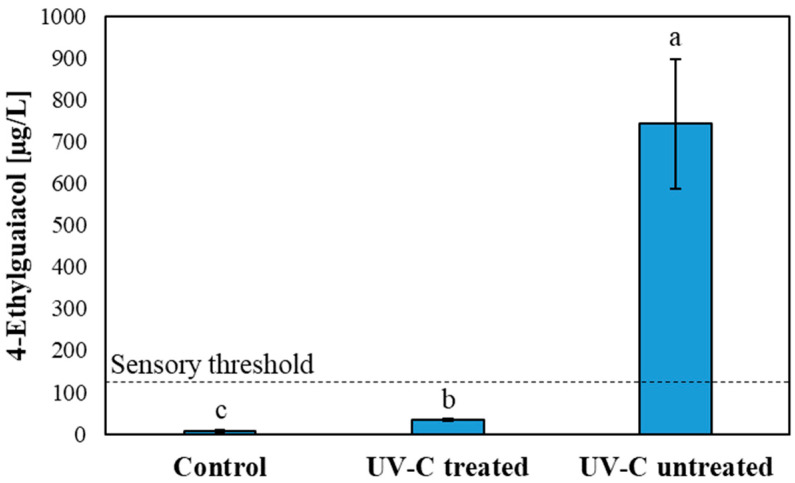
The concentration of 4-ethylguaiacol in Pinot noir wines over a 12-week storage period subjected to different treatments. The data was processed by means of one-way ANOVA. Different letters show differences due to wine variants according to Fisher’s test with *p* ≤ 0.05. Shown are the mean values of experimental replicates (*n* = 3) including the standard deviation.

Figure 1 shows the concentration of 4-ethylguaiacol in Pinot noir wine after a 12-week storage period subjected to different treatments. In the UV-C-treated wine, a significant increase in 4-ethylguaiacol (34 ± 3 µg/L) concentration was observed compared to the control wine (7.3 ± 2.9 µg/L). The concentration of 4-ethylguaiacol did not reach the sensory threshold in red wine (150 µg/L) [6,52]. The microbial results in Section 3.1 showed that *Brettanomyces bruxellensis* was completely inactivated after UV-C treatment and no growth of *Brettanomyces bruxellensis* was detected during the 12-week storage period in UV-C-treated Pinot noir by means of plate counting. The increase in 4-ethylguaiacol in UV-C-treated Pinot noir could have been caused by residual metabolic activity of the enzymes hydroxycinnamic acid decarboxylase and vinylphenol reductase of *Brettanomyces bruxellensis* until cell death. Nevertheless, it cannot be excluded that the formation of 4-ethylguaiacol was caused by the UV-C-induced degradation reaction of ferulic acid. Ferulic acid has an absorption maximum at 254 nm [53], suggesting that ferulic acid is susceptible to photodegradation under exposure to this wavelength, potentially resulting in the formation of 4-ethylguaiacol.

UV-C-untreated Pinot noir wine showed a significantly increased concentration of 4-ethylguaiacol (742 ± 154 µg/L) compared to the control and UV-C-treated wine (Figure 1). These findings are in alignment with the results of the microbial analysis (Section 3.1) indicating sustained microbial activity of *Brettanomyces bruxellensis* in the UV-C untreated wine over the 12-week storage period. The sensory threshold of 4-ethylguaiacol for red wine (150 µg/L) [54] was exceeded almost 5-fold. *Brettanomyces bruxellensis* contains the enzyme hydroxycinnamic acid decarboxylase, encoded by *PAD1*, *DbPAD*, and *DbPAD2* genes [55,56], that catalyzes the transformation of *p*-coumaric, ferulic and caffeic acid into 4-vinylphenol, 4-vinylguaiacol, and 4-vinylcatechol. These vinyl compounds can be transformed through vinylphenol reductase in an anaerobic environment (custer effect) and in the presence of ethanol [6] into 4-ethylphenol, 4-ethylguaiacol, and 4-ethylcatechol, which are associated with the typical *Brettanomyces bruxellensis* off-flavor, such as “animal” and “horse sweat”, in wine [5,57].

Table 2 shows the concentrations of ethyl acetate, ethyl butanoate, 3-methylbutyl acetate, ethyl hexanoate, ethyl octanoate, and ethyl decanoate in Pinot noir wines over a 12-week storage period subjected to different treatments. Esters are secondary aromatic compounds produced during yeast activity are usually associated with alcoholic fermentation by *Saccharomyces cerevisiae* [17] and belong to important aromas that give fresh and fruity notes to wine [58]. Esters are formed through the condensation of an organic acid and an alcohol. The formation is catalyzed by Acetyl-Coenzyme A (Acetyl-CoA) and an esterase [59]. No significant differences could be found between the ester concentrations in control and UV-C-treated Pinot noir wine. These results are in contrast with the increased concentration of 4-ethylguaiacol in UV-C-treated wine as a result of residual metabolic acitivity of the enzymes hydroxycinnamic acid decarboxylase and vinylphenol reductase of *Brettanomyces bruxellensis*, as discussed before. On the one hand, this effect could be caused by direct inhibition of Acetyl-Coenzyme A and/or esterase through UV-C. In fact, UV-C treatment can inhibit certain enzymes through direct damage, free radical formation, or changes in protein structure [60,61]. On the other hand, the unchanged ester concentrations in UV-C-treated wine could be a result of opposing reactions. If any residual enzymatic activity of *Brettanomyces bruxellensis* was forming esters [59,62], parallel degradation of esters through UV-C could have decreased their concentration, eventually resulting in unchanged ester concentrations in UV-C-treated wine [42,44]. Overall, the results do not indicate microbial activity of viable *Brettanomyces bruxellensis* cells in the UV-C-treated wine during the 12-week storage period. These results are consistent with microbial examination and underline the long-term effectivity of UV-C treatment for the microbial stabilization of wine and are in agreement with the study by Junqua et al. [35], who showed no significant changes in aroma-active substances of UV-C-treated red wine after two years of storage.

The UV-C untreated wine showed a significant increase in the concentration of all measured ester compounds (Table 2). The formation of esters during the storage period can be attributed to the metabolic activity of viable *Brettanomyces bruxellensis* cells [63]. These results are consistent with the results of the microbial examination (Section 3.1) and confirm the microbial activity of *Brettanomyces bruxellensis* in UV-C untreated wine during the storage period of 12 weeks.

### 3.3. Phenolic Compounds

Table 3 shows the concentrations of catechin, gallic acid, caftaric acid, *p*-coumaric acid, and ferulic acid in Pinot noir wines over a 12-week storage period subjected to different treatments. While catechin and gallic acid concentrations were not affected by UV-C tretament, a significant decrease in the concentration of caftaric acid and *p*-coumaric acid could be detected in the UV-C-treated wine compared to the control wine after the 12-week storage period. The decrease in *p*-coumaric acid after UV-C treatment can be explained by the residual metabolic activity of the spoiled microorganism until its cell death [57]. Since caftaric acid was shown not to be a substrate of *Brettanomyces bruxellensis* [64], its decrease after UV-C treatment may be solely attributed to a photochemical reaction. In fact, the authors of [65] showed that tartaric esters of hydroxycinnamic acids are susceptible to light-induced reactions. One absorption maximum of caftaric acid is in the area of UV-C treatment at 254 nm [66]; accordingly, it can be excited under UV-C light, potentially be transformed to a quinone, and eventually undergo further reactions. The concentration of caftaric acid in the UV-C untreated and control wine revealed no differences, confirming the postulates of Schopp et al. [64] in that caftaric acid is not a substrate of *Brettanomyces bruxellensis*. However, *p*-coumaric acid showed a significant decrease in the UV-C untreated wine compared to the UV-C-treated and control wine. This confirms the microbial activity of *Brettanomyces bruxellensis* in the wine during the 12-week storage period. The concentration of ferulic acid was below the LOQ in all treatments. This suggests that the compound may have been present at trace levels, but accurate quantification was not possible due to the limited sensitivity and calibration linearity of the method at low concentration ranges.

## 4. Conclusions

This study demonstrates that the application of the UV-C technique not only inactivates *Brettanomyces bruxellensis* but also facilitates the microbial storage stability of wine. During the 12-week storage period after UV-C treatment at 24 kJ/L, no increase in the cell count of *Brettanomyces bruxellensis* was observed in Pinot noir. No significant differences in the concentrations of aroma-active compounds between the control and UV-C-treated wine could be detected, except for a marginal increase in 4-ethylguaiacol due to the residual metabolic activity of *Brettanomyces bruxellensis*. A significant decrease in caftaric acid concentration was observed in UV-C-treated wines and attributed to oxidative reactions induced by UV-C exposure. While these findings indicate that UV-C treatment is effective in allowing microbial stability and preserving wine quality, the exact mechanisms of the residual metabolic activity of *Brettanomyces bruxellensis* forming 4-ethylguaiacol after UV-C treatment remain unclear. In particular, studies are needed to determine whether sublethal microbial activity or non-enzymatic oxidative pathways contribute to this phenomenon of the formation of 4-ethylguaiacol and other volatile phenols. Furthermore, while this study focused on a 12-week storage period, it would be valuable for the authors of future research to investigate the longer-term impact of UV-C treatment on wine composition and sensory properties. Previous work [35] has shown that UV-C-induced changes in esters, polyphenols, or color may only become apparent after several months of bottle aging. Integrating extended storage evaluations and time-resolved sensory analysis would offer a more comprehensive understanding of UV-C treatment’s suitability for red wines intended for long-term aging. The authors of future research should also consider including a treatment group exposed to UV-C in the absence of *Brettanomyces bruxellensis*, in order to distinguish between microbial and UV-C-induced chemical changes in wine. Furthermore, sensory evaluation should be integrated into upcoming studies to better assess the potential impact of UV-C treatment on the organoleptic properties of wine.

## Figures and Tables

**Table 1:** Cell count of *Brettanomyces bruxellensis* in Pinot noir wines over a 12-week storage period subjected to different treatments. The data was processed by one-way ANOVA. Different letters show significant differences according to Fisher’s test with *p* ≤ 0.05; LOQ: limit of quantification. Shown are the mean values of three experimental and three analytical replicates (*n* = 3 × 3) including the standard deviation.

Week	Control [CFU/mL]	UV-C-Treated [CFU/mL]	UV-C Untreated [CFU/mL]
0	˂LOQ	˂LOQ	3.6 × 10^5^ ± 4.0 × 10^4^ d
2	˂LOQ	˂LOQ	3.7 × 10^5^ ± 4.0 × 10^4^ d
4	˂LOQ	˂LOQ	9.0 × 10^5^ ± 3.0 × 10^4^ c
6	˂LOQ	˂LOQ	9.3 × 10^5^ ± 5.0 × 10^4^ c
8	˂LOQ	˂LOQ	1.4 × 10^6^ ± 4.0 × 10^4^ a
10	˂LOQ	˂LOQ	1.2 × 10^6^ ± 8.0 × 10^4^ b
12	˂LOQ	˂LOQ	9.4 × 10^5^ ± 7.0 × 10^4^ c
*p*-value	n.d.	n.d.	˂0.0001

**Table 2:** The concentration of volatile compounds of the aroma class ester in Pinot noir wines over a 12-week storage period subjected to different treatments. The data was processed by means of one-way ANOVA. Different letters show differences due to wine variants according to Fisher’s test with *p* ≤ 0.05; LOD—limit of detection. Shown are the mean values of experimental replicates (*n* = 3) including the standard deviation.

	Ethyl Acetate [mg/L]	Ethyl Butanoate [µg/L]	3-Methylbutyl Acetate [µg/L]	Ethyl Hexanoate [µg/L]	Ethyl Octanoate [µg/L]	Ethyl Decanoate [µg/L]
Control	35 ± 4 a	151 ± 17 a	54 ± 8 a	155 ± 20 a	117 ± 18 a	<LOD
UV-C-treated	33 ± 3 a	142 ± 16 a	58 ± 4 a	154 ± 16 a	118 ± 19 a	<LOD
UV-C-untreated	57 ± 7 b	213 ± 23 b	594 ± 57 b	231 ± 38 b	242 ± 39 b	36 ± 10
*p*-value	˂0.0001	0.0002	˂0.0001	0.0002	˂0.0001	n.d.

**Table 3:** The concentrations of catechin, gallic acid, caftaric acid, p-coumaric acid, and ferulic acid in Pinot noir wines over a 12-week storage period subjected to different treatments. The data was processed by means of one-way ANOVA. Different letters show differences due to wine variants according to Fisher’s test with *p* ≤ 0.05. Shown are the mean values of experimental replicates (*n* = 3) including the standard deviation.

	Catechin [mg/L]	Gallic Acid [mg/L]	Caftaric Acid [mg/L]	*p*-Coumaric Acid [mg/L]	Ferulic Acid [mg/L]
Control	20 ± 3 a	16 ± 2 a	50 ± 1 a	1.3 ± 0.1 a	˂LOQ
UV-C-treated	19 ± 2 a	15 ± 1 a	44 ± 2 b	1.0 ± 0.1 b	˂LOQ
UV-C-untreated	19 ± 1 a	17 ± 1 a	52 ± 2 a	0.8 ± 0.1 c	˂LOQ
*p*-value	0.813	0.296	0.003	0.0025	n.d.

## Data Availability

The original contributions presented in this study are included in the article. Further inquiries can be directed to the corresponding author.
